# Supplementary material for: The interactome of intact mitochondria by cross-linking mass spectrometry provides evidence for coexisting respiratory supercomplexes
Source: Mol Cell Proteomics. 2017 Dec 8;17(2):216–32. doi: 10.1074/mcp.RA117.000470 (PMC5795388; doi:10.1074/mcp.RA117.000470)
Supplement: Supplemental Data [file supp_RA117.000470_133922_0_supp_23978_fzffwf.zip › spectra_annotation/mito_DR_spectra_annotation/182-1-10-1-1-1.pdf]

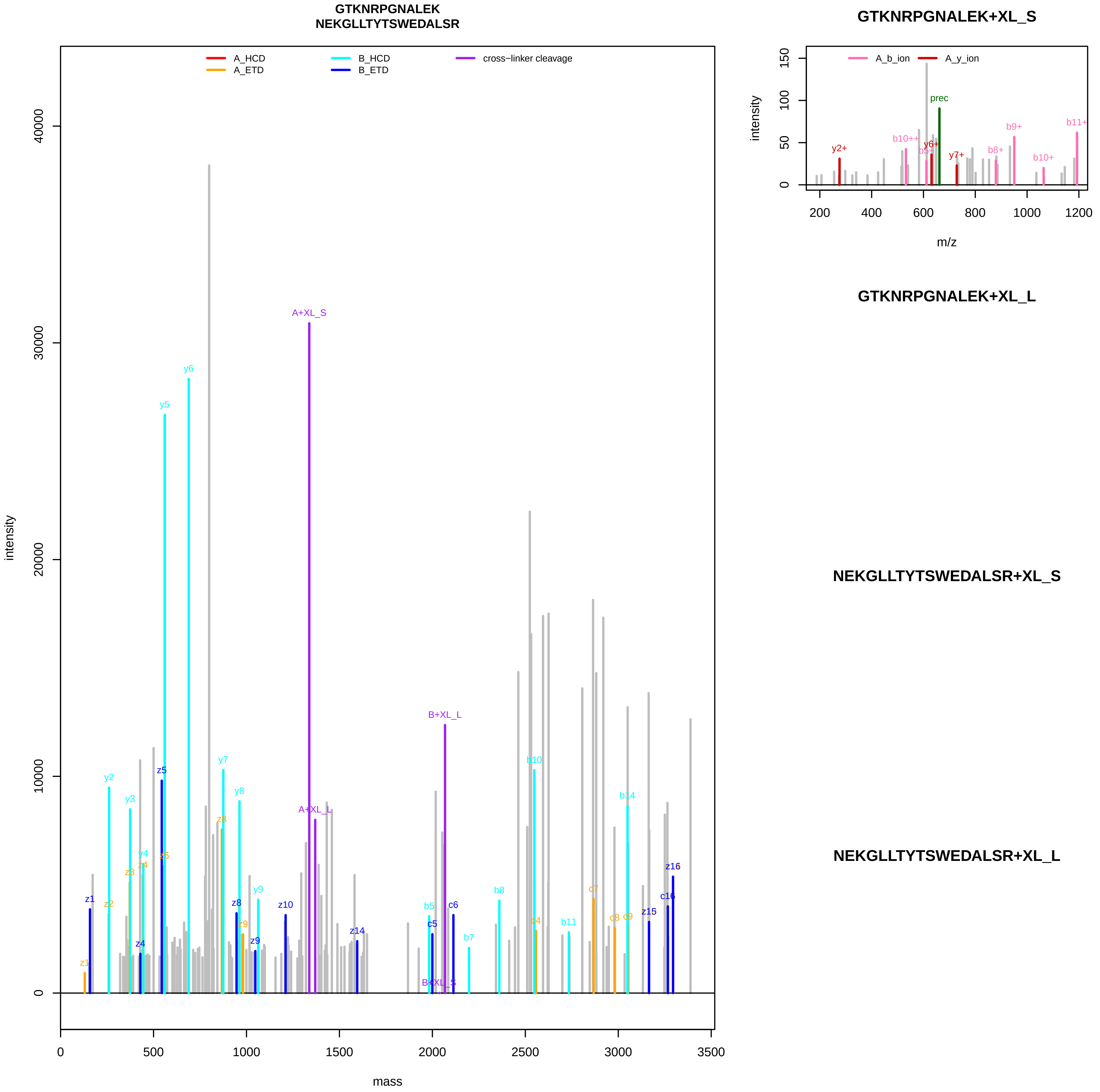
GTKNRPGNALEK
NEKGLLTYTSWEDALSR
A_HCD
A_ETD
B_HCD
B_ETD
cross−linker cleavage
intensity
mass
0
10000
20000
30000
40000
0
500
1000
1500
2000
2500
3000
3500
A+XL_S
B+XL_L
A+XL_L
B+XL_S
y2
y3
y4
y5
y6
y7
y8
y9
z1
z2
z3
z4
z5
z8
z9
z10
z14
z15
z16
b5
b7
b8
b10
b11
b14
c4
c5
c6
c7
c8
c9
c16
GTKNRPGNALEK+XL_S
A_b_ion
A_y_ion
intensity
m/z
0
50
100
150
200
400
600
800
1000
1200
prec
y2+
y6+
y7+
b5+
b8+
b9+
b10++
b10+
b11+
GTKNRPGNALEK+XL_L
NEKGLLTYTSWEDALSR+XL_S
NEKGLLTYTSWEDALSR+XL_L
